# Co-Designing a Digital Brief Intervention to Reduce the Risk of Prescription Opioid–Related Harm Among People With Chronic Noncancer Pain: Qualitative Analysis of Patient Lived Experiences

**DOI:** 10.2196/57208

**Published:** 2025-01-30

**Authors:** Rachel A Elphinston, Sue Pager, Kelly Brown, Michele Sterling, Farhad Fatehi, Paul Gray, Linda Hipper, Lauren Cahill, Jason P. Connor

**Affiliations:** 1 RECOVER Injury Research Centre The University of Queensland Herston Australia; 2 National Health and Medical Research Council Centre for Research Excellence - Better Health Outcomes for Compensable Injury The University of Queensland Brisbane Australia; 3 School of Psychology The University of Queensland Brisbane Australia; 4 Metro South Addiction and Mental Health Services Metro South Health Hospital and Health Service Brisbane Australia; 5 Health Equity & Access Unit Metro South Hospital and Health Service Brisbane Australia; 6 Gallipoli Medical Research Foundation Greenslopes Private Hospital Brisbane Australia; 7 Centre for Online Health The University of Queensland Brisbane Australia; 8 School of Psychological Sciences & Turner Institute for Brain and Mental Health Monash University Melbourne Australia; 9 Tess Cramond Pain and Research Centre Metro North Hospital and Health Service Brisbane Australia; 10 Faculty of Medicine The University of Queensland Brisbane Australia; 11 National Centre for Youth Substance Use Research The University of Queensland Brisbane Australia

**Keywords:** chronic noncancer pain, prescription opioid use, brief intervention, co-design, patient partners, lived experience, qualitative, digital health

## Abstract

**Background:**

Opioid medications are important for pain management, but many patients progress to unsafe medication use. With few personalized and accessible behavioral treatment options to reduce potential opioid-related harm, new and innovative patient-centered approaches are urgently needed to fill this gap.

**Objective:**

This study involved the first phase of co-designing a digital brief intervention to reduce the risk of opioid-related harm by investigating the lived experience of chronic noncancer pain (CNCP) in treatment-seeking patients, with a particular focus on opioid therapy experiences.

**Methods:**

Eligible patients were those aged between 18 and 70 years with CNCP at a clinically significant level of intensity (a score of ≥4 of 10). Purposive sampling was used to engage patients on public hospital waitlists via mail or through the treating medical specialist. Participants (N=18; n=10 women; mean age 49.5 years, SD 11.50) completed semistructured telephone interviews. Interviews were transcribed verbatim, thematically analyzed using grounded theory, and member checked by patients.

**Results:**

Eight overarching themes were found, listed in the order of their prominence from most to least prominent: limited treatment collaboration and partnership; limited biopsychosocial understanding of pain; continued opioid use when benefits do not outweigh harms; a trial-and-error approach to opioid use; cycles of hopefulness and hopelessness; diagnostic uncertainty; significant negative impacts tied to loss; and complexity of pain and opioid use journeys.

**Conclusions:**

The findings of this study advance progress in co-designing digital brief interventions by actively engaging patient partners in their lived experiences of chronic pain and use of prescription opioid medications. The key recommendations proposed should guide the development of personalized solutions to address the complex care needs of patients with CNCP.

## Introduction

Opioid therapy is recommended as part of a multimodal approach to manage acute, postoperative, and cancer pain [1]. There is considerably less evidence on the effectiveness of opioid therapy for patients with chronic noncancer pain (CNCP) [2,3], and meta-analyses show modest CNCP relief [4]. The risks of adverse side effects [5], including opioid-related overdose death [6,7], remain a major public health concern [8-11], especially as (1) access to interdisciplinary rehabilitation is restricted by cost, treatment intensity, geography, demand [12-16], and patient fears about opioid discontinuation [17-19] and (2) the effectiveness of behavioral and pharmacological treatments to reduce the risks of opioid use in patients with CNCP is inconclusive [20,21].

Digital brief interventions (BIs) may offer a nonpharmacological option to reduce opioid-related harm [22,23]. BIs are widely used in substance use treatment, and they involve screening, feedback assessment, education, and goal setting, motivating those at risk to modify substance use and engage in further treatment if required [24]. BIs are effective in reducing substance use and related harm in various settings (eg, primary care and emergency departments) [25,26] and have shown similar effectiveness to more intensive psychological therapies [27,28]. Digital BIs are becoming an increasingly attractive, scalable technological innovation in the management of pain that may be just as effective as in-person BIs in reducing substance use and related harm [29]. The use of digital BIs in the CNCP population is still in its infancy, but there is interest among patients in these types of interventions [23,30].

Broadly speaking, as effective as digital health interventions (DHIs) are, they are not widely adopted in practice [31,32]. One of the main reasons is that key stakeholders (eg, patients and health professionals) have not been a part of the solution design [33-36]. Research co-design, that is, the meaningful involvement of end users in the design, implementation, and translation of research, has the potential to reduce the risk of research failing to translate into practice [37]. Co-design offers a means to increase the success of DHIs by aligning with user needs and preferences [38,39].

Co-design of DHIs for CNCP has not made significant progress over the past decade [40]. Most DHIs for pain or medication use are developed by the for-profit software industry [41], lack a theoretical framework [42] and evidence base [43]. Only 19% of studies involve end users in the development process, and this is usually in an ad hoc manner [44]; few studies support clinician access or involvement [42,44,45].

Using a co-design approach with patient partners makes it possible to design a fit-for-patient-purpose digital BI, which may accelerate its adoption and impact considerably. The first step of the co-design of DHIs is to obtain a rich and deep understanding of people’s experiences within the disease management context while also sensitizing them to the problem space [46,47]. Despite the diverse and unique lived experiences of those with CNCP (eg, [48,49]), DHIs are rarely designed in partnership with this patient population. This study aimed to understand the lived experiences of patients with CNCP with a particular focus on opioid therapy—the first important step of co-design. The patients were informed of the overall goal to co-design a digital BI and then asked to describe their experiences of CNCP (past, present, and future) management, including opioid therapy. This new knowledge will inform future steps in the co-design process of digital BIs for the population that is most at risk of harmful use of opioid medicines.

## Methods

### Participants

Participants were eligible if they were aged between 18 and 70 years, living with CNCP (defined as a sensory and emotional experience that lasts or recurs for ≥3 months), and seeking treatment from public health specialist addiction or pain services. Participants were also required to report clinical levels of pain, as indicated by scoring ≥4 out of 10 on the Pain Numerical Rating Scale on average over the past week.

Participants were excluded if they were assessed as being highly distressed (based on scores ≥13 on the Kessler Psychological Distress Scale [50] and follow-up telephone risk assessments by the research team psychologists [RE and KB]); were non–English speaking; had a history of recent injecting drug use; or had currently tested positive with SARS-CoV-2. In addition, patients recruited from addiction services were excluded based on the clinical judgment from the addiction specialist if they had an acute, severe mental illness (eg, active psychosis).

### Design

**Figure 1:**
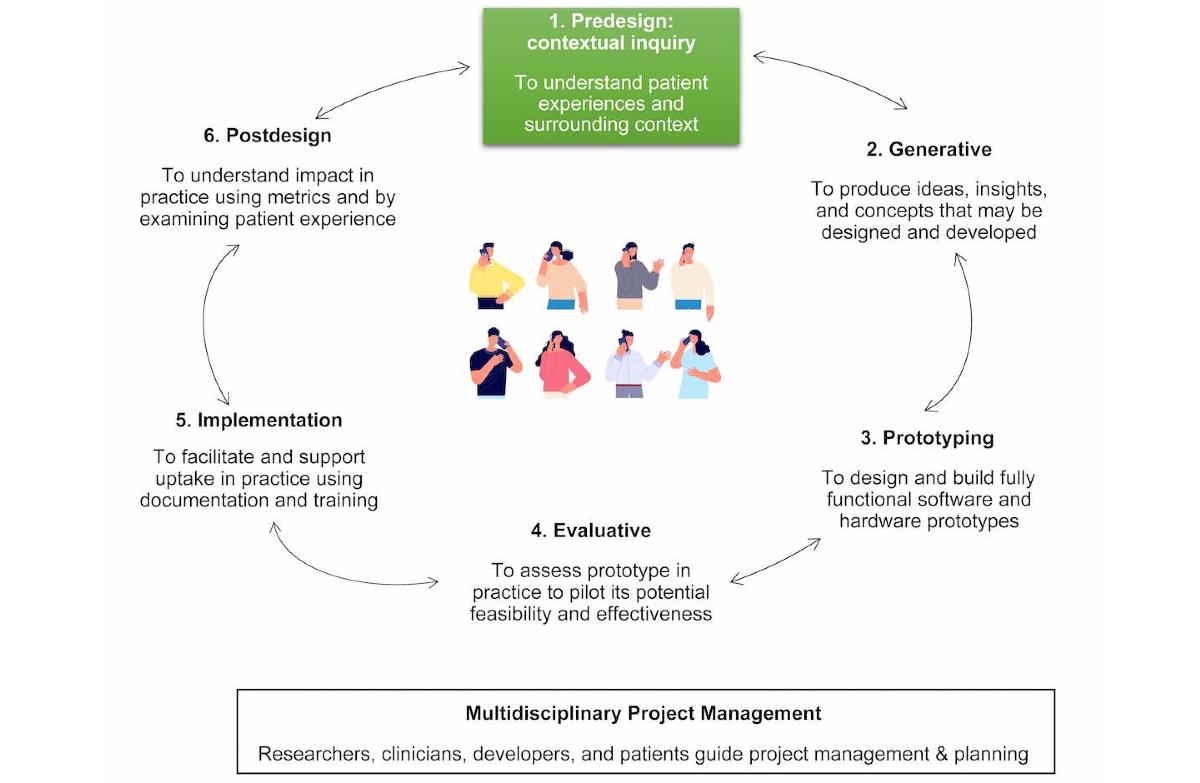
Overview of the iterative co-design framework used in this study. The predesign phase, denoted by the box highlighted in green, is reported in this study.

A co-design approach [51] was used. We applied the co-design framework by Sanders and Stappers [47] and updated by Noorbergen et al [46] during the co-design of the digital BI. It outlines 6 iterative co-design phases: predesign, generative, prototyping, evaluative, implementation, and postdesign. Figure 1 presents an overview of the co-design framework. This study reports on the first phase of the iterative co-design development process – the exploration of patient experiences as part of predesign (ie, contextual inquiry). A systematic review examining the state of the evidence and an adjunctive study examining patient preferences for BIs to reduce pain, opioid use, and related harm was also conducted as part of the first phase and is reported elsewhere [23].

### Recruitment

Treatment-seeking patients who were currently using prescription opioids or had a history of opioid use were purposively sampled. Participants with CNCP were recruited through a tertiary hospital specialist pain clinic or community addiction specialist service. Patients from the pain clinic were engaged by mailing an information pack inviting those on the waitlist to participate. Participants from the hospital addiction service were invited by the medical specialist and a member of the research team (RE) during their on-site appointment at the clinic. The final sample size was determined by thematic saturation using the approach outlined by Guest et al [52], applied during the data collection and inductive thematic analysis phase using 3 components: base size, run length, and new information threshold. The base size refers to the body of information identified in the initial dataset (ie, initial number of themes) and is used as a denominator of the saturation ratio. The run length refers to the number of interviews or other data collection events based on which new information or themes are identified. New information threshold refers to the level of information accepted as indicative of saturation. A new information threshold of ≤5% was selected to indicate that we had reached adequate saturation.

### Procedure

**Figure 2:**
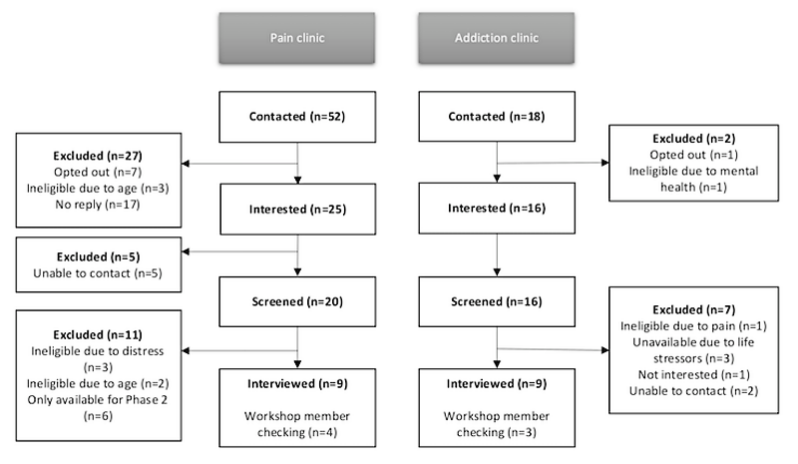
Patient flow.

Recruitment and interviews were completed from June to November 2020. Participants completed an initial telephone screening interview for us to determine eligibility. If eligible, patients completed a web-based questionnaire before the interview for descriptive purposes and to provide background to guide the interview. Interviews were audio-recorded, professionally transcribed verbatim, and thematically analyzed. Member checking of themes by patients who participated in the interviews was conducted during a follow-up workshop, which focused on the design components of the BI (not reported in this study). Figure 2 presents a summary of patient flow.

### Measures

#### Demographics

Demographic data, including age, sex, and cultural background, as well as information on pain location, duration, intensity, and current medications were collected. Data on history of mental health or substance use disorder were also collected.

#### Brief Pain Inventory

The Brief Pain Inventory [53,54] is an 8-item measure that assesses the pain experience, including location of pain, pain intensity and interference on a scale from 0 (no pain) to 10 (pain as bad as you can imagine). Scores are summed to produce 2 separate scores of pain intensity and pain interference, with higher levels indicating greater intensity and interference. An example item is as follows: “Please rate your pain by circling the one number that best describes your pain at its worst in the last week.” The Brief Pain Inventory is a validated and widely used measure in clinical settings with efficacy in both patients with cancer pain and those with chronic, noncancer pain [53,54].

#### Kessler Psychological Distress Scale

The Kessler Psychological Distress Scale [50] is a 6-item measure used to assess a person’s level of psychological distress. Participants were asked how often they have felt a range of emotions over the past 4 weeks (eg, “How often did you feel anxious?”). Responses are scored on a 5-point scale (0=none of the time; 4=all of the time). Scores ≥13 indicate the presence of a possible mental illness [50]. This scale has demonstrated excellent reliability [50].

#### Depression, Anxiety, and Stress

The 21-item Depression Anxiety Stress Scales [55] asked participants to rate to what degree depressive, anxiety, or stress symptoms have impacted them in the previous week on a scale from 0 (did not apply to me at all) to 3 (very much or most of the time). Items from each subscale were summed and multiplied by 2 to denote a patient’s current level of depression, with higher scores reflecting higher levels of depression. An example item from the depression subscale is as follows: “I felt that I had nothing to look forward to.” The 21-item Depression Anxiety Stress Scales has previously been shown to be a reliable and valid tool in a chronic pain sample (Cronbach α=0.96 for depression, Cronbach α=0.89 for anxiety, and Cronbach α=0.95 for stress) [56].

#### Current Opioid Misuse Measure

The Current Opioid Misuse Measure (COMM) [57] consists of 17 items measured on a 0 (never) to 4 (very often) scale, which asks patients about their current opioid use behaviors. Scores are summed, with higher totals reflecting higher levels of opioid misuse. An example item is as follows: “In the past 30 days, how often have you taken your medication differently to how they are prescribed?” The COMM is a validated and reliable measure within chronic pain samples, with previous research reporting Cronbach α=0.86 [57-59].

### Semistructured Interview

Semistructured interview questions explored patient experiences and their initial perspectives of a digital BI (Multimedia Appendix 1). Participants were first informed about the overall aim of the research, to co-design a digital BI to reduce risk of unsafe use of prescription opioid medications. They were then asked about their past, current, and future experiences of pain and pain management; access to treatment services and programs including psychologists and psychological treatment; and specifically, their experience of using prescription opioids. Then, suggestions for the design and implementation of the digital BI were explored; however, these will be reported in a separate paper as part of the second (generative) phase of the co-design process [46,47]. All interviews were conducted by RE, a clinical psychologist, and KB, a psychologist, both with expertise in treatment of chronic pain and qualitative research. Field notes were made after the interviews to record the interviewer’s observations and impressions.

### Data Analysis

Transcripts were thematically analyzed using grounded theory [60]. Two independent coders (RE and SP) read the transcripts to establish familiarity. An inductive approach to coding was used, with codes added as they were identified in the data. The coding structure consisted of first level codes recorded line-by-line, second level codes of possible subthemes, and third level codes of higher order themes. Key quotes were extracted. The analysis was conducted iteratively, with codes cross-checked between coders approximately every 3 to 5 transcripts to identify any discrepancies, which were resolved by further discussion. Theme refinement was undertaken between the coders and another interviewer (KB), integrating the interviewer’s field notes to aid interpretation and reduce coder bias.

Member checking [61] was conducted in a subsample (n=7) of interviewed participants in subsequent workshops to explore the validity of the themes as part of the assessment of data saturation approach, that is, to check whether the themes were consistent with the participant’s experience. This involved facilitators providing verbal feedback on the themes integrated with the workshop activities. We sought disconfirming voices (objectivism) and provided opportunities for reflection on personal experiences, creating opportunities to add data (constructivism) [62].

### Ethical Considerations

Ethics approval was obtained from the relevant institutional review boards (HREC/2020/QMS/60695). All participants provided written informed consent. Study data were deidentified for analysis, and any identifying information was removed before publication, including patient numbers, to ensure complete anonymity. All participants were offered an Aus $50 (US $33) gift voucher for each session attended to thank them for their time.

## Results

### Data Saturation Analysis

Table 1 presents a summary of the data saturation analysis. We reached adequate saturation (≤5% threshold) after 16^+2^ in-depth interviews. The final 2 extra interviews did not add substantially to the body of information collected; however, these ensured a purposive sampling approach included a balance of male and female participants and people from various culturally and linguistically diverse backgrounds.

**Table 1:** Data saturation analysis.

Step	Outcome
1. Number of themes for the base interviews^a^	8 × PID_P^b^ interviews
2. Number of unique themes for the first run	5 new themes from 4 × PID_A^c^ interviews
3. Saturation ratio	Number of new themes/run: 5/30 = 17% (>5% threshold); continue with interviews
4. Number of new, unique themes for the next run	2 new themes from 5 × PID_A interviews and interviewer reflections: 2/30=7%
5. Updated saturation ratio	7% (>5% threshold); continue with interviews
6. Number of new unique themes for the next run	1 new theme from 2 × workshop member checking: 1/30=3%
7. Updated saturation ratio	3%, less than ≤5% threshold
8. Number of new unique themes for the next run (to address gaps in demographics)	1 new theme from 1 × PID_A and 1 × PID_P interview: 1/30=3%
9. Updated saturation ratio	3%, ≤5% threshold

^a^Denominator in the equation.

^b^PID_P: patients from the pain clinic.

^c^PID_A: patients from specialist addiction services.

### Sample Characteristics

The sample included 10 women and 8 men, with a mean age of 49.5 (SD 11.50; range 25-62) years. One-third of the patients self-identified an ethnicity in addition to Australian descent, including 2 from Aboriginal and Torres Strait Islander descent. Four (22%) patients were employed either part time or full time. Approximately half of the participants reported their highest level of education as grade 12 and 4 (22%) participants had at least an undergraduate degree. More than half of the patients were in a relationship.

The sample predominantly reported back or neck pain (89%). Duration of pain ranged from 6 months to 42 years (mean 11.57, SD 11.55 years; median 7 years). Average pain severity in the past week was 5.82 (SD 1.51), and average pain interference in the past week was 5.80 (SD 1.91). In total, 16 (89%) patients were currently using prescription opioids (range 1.15 mg/d [pro re nata] to 480 mg/d), and 2 (11%) patients had used opioid therapy in the past. Half of the participants reported current opioid misuse (scores ≥9 on the COMM) [57]. Three (19%) patients were prescribed high-dose opioids (≥100 mg/d) [63]. Seven (39%) patients reported moderate depressive symptoms. Three (17%) reported severe or extremely severe depressive symptoms; these patients were included as they were assessed to have adequate supports and risk management plans, which supported their involvement. Table 2 presents a summary of individual characteristics (see Multimedia Appendix 2 for a more detailed summary of individual patient characteristics). 

**Table 2:** Sample characteristics of patients.

Age (y)	Sex	Pain duration (y)	Pain interference (past week)	Pain severity (past week)	COMM^a^	OME^b^
33	Female	15	2.71	2.25	4	1.15
52	Male	2	9.14	7	5	30
60	Female	3	5.00	6	7	45
31	Male	5	4.14	5	14^c^	60
47	Female	0.5	4.71	6	—^d^	—^d^
61	Female	4	2.86	5	8	—^d^
53	Female	14	4.71	4	10^c^	70
47	Female	0.83	6.57	6.75	20^c^	22.8
58	Female	42	8.86	8	13^c^	143.4
60	Female	2.5	6.43	7.25	2	37.9
54	Male	20	7.43	7.25	25^c^	480
25	Male	3	5.83	6.5	1	4.2
58	Male	11	6.43	5.5	15^c^	N/A^e^
53	Male	20	5.29	5.75	13^c^	N/A^e^
56	Female	28	8.00	6.75	3	N/A^e^
48	Male	9	4.14	6.5	11^c^	90
33	Female	3.5	4.29	3	27^c^	480
62	Male	25	7.71	6.25	16^c^	20

^a^COMM: Current Opioid Misuse Measure.

^b^OME: oral morphine equivalence (daily).

^c^These scores meet the cutoff for the scale as risk of unsafe opioid use.

^d^Missing.

^e^N/A: not applicable; patients were using methadone and dose could not be calculated.

### Thematic Analysis

#### Overview

**Figure 3:**
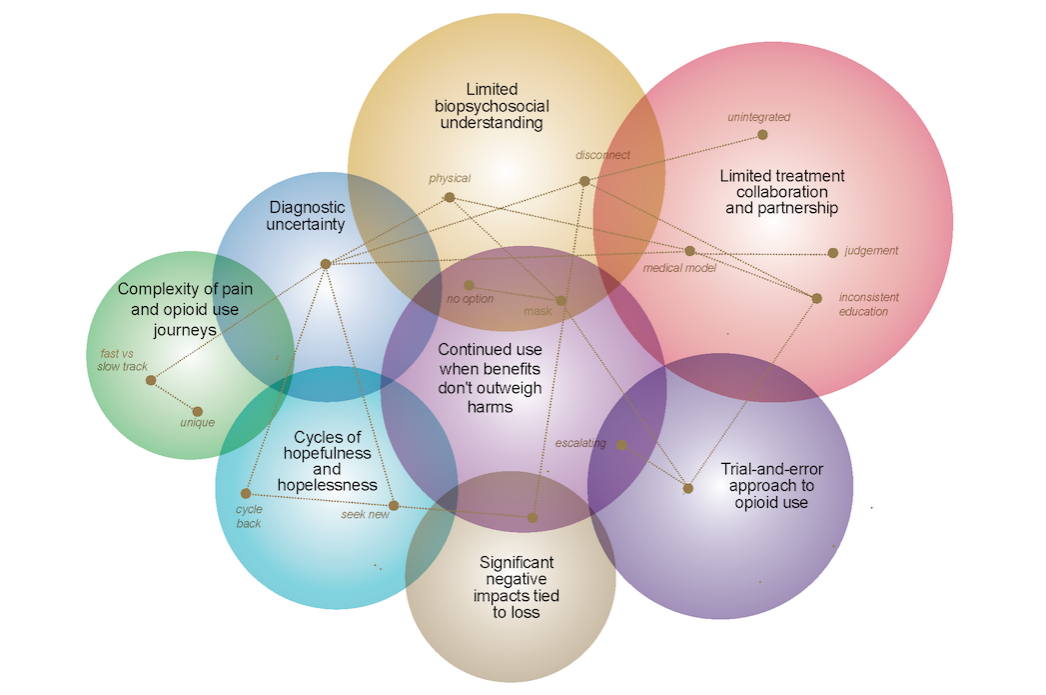
Overview of themes and subthemes. The overlap of circles represents connections between themes, and lines represent connections between subthemes.

Eight overarching themes and 13 subthemes were identified from the data, listed in the order of prominence from most to least prominent: limited treatment collaboration and partnership; limited biopsychosocial understanding of pain; continued opioid use when benefits do not outweigh harms; a trial-and-error approach to opioid use; cycles of hopefulness and hopelessness; diagnostic uncertainty; significant negative impacts tied to loss; and complexity of pain and opioid use journeys. Figure 3 provides an overview of the themes; larger circles reflect more prevalent themes. In subsequent sections, note that PID_P refers to patients from the pain clinic and PID_A refers to patients from addiction services. Patient numbers were removed before publication to ensure complete anonymity.

#### Theme 1: Limited Collaboration and Partnership During Treatment

This theme reflected the lack of a patient–health professional collaborative partnership and an integrated approach among health professionals in the care of patients.

##### Patients Initially Followed the Medical Model, With Faith and Trust

Initial opioid prescription was usually prompted by the treating physicians in response to an acute episode of pain or following surgery. Patients with a long history of pain described early prescribing practices as including more passive rather than active involvement of patients. One patient (PID_P) reported the following:

So the Dr says, look here, take this, this will stop the pain. So, you go, ok, you take it.

Patients said they often continued to follow the medical model, with faith and trust in the treatment approach guided by their physician. This suggested a lack of shared decision-making about whether opioids were the best or right treatment option and whether the benefits outweighed potential harms—“I’m in the hands of the Doctors” (PID_A). As their pain persisted over time, trust in opioid or medical treatment decreased and participants tried to seek alternatives treatments to manage pain. Some patients reported remaining very trusting of their general practitioner (GP) and were more actively involved in discussing options with them. Others reported becoming distrustful and even angry.

##### Inconsistent Opioid Use Education, Monitoring, and Support

There was variation in the amount of education about opioid use that the patients received when first prescribed. Some patients said they received education from their physician or pharmacist about the potential harmful effects of prescription opioids (eg, side effects and addiction potential). Other participants could not recall receiving any education in this regard:

A lot of doctors don’t have communication or you know, they’ll just walk in, they’ll give you the script and walk out. But I think it’s very important for the doctors, as the ones prescribing to actually sit there and go, all right, here’s the side effects.

PID_A

The reported level of ongoing education and medical monitoring of opioid use over time also varied. One patient (PID_P) suggested, “They should have an opiate diary and they should make people keep diaries of when they take them and how many, that would allow them to monitor usage because I get given a prescription, but nobody actually monitors my usage.”

Patients also described the discussions they had with their physician about possible changes to their opioid use. Some patients felt that doctors prescribed opioids when they were not sure about or had exhausted other treatment options. For example, one patient (PID_P) reported, “I have tried to ask my doctor is there anything else I can try to see if it works better...his advice was there’s not really anything else that’s going to be able to help.” Lack of education appeared to fuel questions later during treatment. Some patients questioned why they were still using opioids, given that they initially expected opioids to be a relatively short-term solution for pain: “I personally thought this (opioids) might be taken 6-8 months maybe a year, we’ll fix it, back to work” (PID_P). One patient said that doctors had different opinions and that the tapering plan differed to information on the internet, so they did not progress with it. Some reported being encouraged to taper in the context of recent policy changes to accessing opioids but did not always feel they had adequate support to do this. One patient (PID_A) said the following:

You know, and they keep saying to you try and cut down, you try and cut down so much. You’re in pain, cutting down, you show that you can try and cut down.... I know I don’t want to be on them, I should be off them by now if it had of gone right. I think you just feel isolated because we don’t have anybody we can really talk to about our pain anymore.

Some patients described that they had not discussed tapering with their physician as they were worried that pain may increase and were fearful of the physician’s response. One patient (PID_P) said the following:

I was too scared to say to him “look I want to start reducing” because I just thought if I couldn’t cope with the pain on a reduced dose he many not let me go back up again and then I would be stuck with pain. So that stopped me sort of officially asking for help.

##### Health Professional and System Judgment, Distrust, and Stigma

This theme related to the patients’ general feeling of disillusionment and distrust of mainstream medicine because of continued pain and seeking to apportion blame (eg, “I don’t trust doctors. I don’t trust physios. I don’t trust anybody because of what they’ve done to me” [PID_A]). When patients described more active attempts to discuss pain relief, concerns about addiction reportedly impacted the clinician’s decision to prescribe opioid medications. One patient (PID_P) stated the following:

Drs are more bothered about other people’s addiction rather than prescribing me pain relief.... My pain was not her concern when I went and asked for strong pain killers.

These reports of mistrust were also observed when patients discussed the impact of recent policy changes in Australia that restricted access to over-the-counter codeine medicines (February 2018) and medications for CNCP (June 2020). There was an understanding that the system and the message had changed, leaving them in a difficult position: “well the medical community’s clearly been saying for a long time ‘you need to take this, you need to take this’, and then they say ‘well no, you’re not allowed to take them anymore’, you’re left with ‘well, nothing else works, what are you going to do?’” (PID_A). For patients from addiction services, there were cases where their physician had ceased prescription, which prompted referral to an addiction specialist and engagement in an opioid replacement program. This was accompanied by feelings of abandonment toward their previous prescriber and, broadly, the medical system and ambivalence about accessing an addiction service. For some, this led to consideration of other treatment alternatives, including psychological therapy and cannabis (both prescribed and nonprescribed).

Several patients reported feeling increasingly sensitive to questioning and comments about their opioid use and chronic pain condition:

She would sort of say well what medications are you on? Oh, that’s a high dose. oh that’s been a long time. I feel like they make you feel like you’re doing the wrong thing but nobody is offering any sort of solutions.

PID_P

Some patients reported finding this confronting and exhibited some defensiveness to the possibility of addiction or dependency. One patient (PID_A) said, “So I don’t think I was addicted to it, I just took it because I got pain.”

This appeared to contribute to the possible stigma associated with addiction more broadly. A patient (PID_A) explained as follows:

My doctor sent me there.... It kind of made me feel like I had a habit or had an addiction. That kind of really put me on the spot, and I didn’t really want to go. But I went anyway. So if you put someone that’s willing to cut down, but then you send them to a place like that they might feel attacked in a certain way. Like they’re in the same category as those people but they’re trying to do it for good reasons, not because they’re addicts.

One patient (PID_A) also described being on methadone as stigmatizing as “only heroin users use methadone.” Discussions indicated that some patients felt that addiction services were not the right place for them to seek treatment and that they were *different* from other drug users. There was also a difference for people between acknowledging that they may be addicted to opioids versus the label of “addict” or “junkie.” It is possible that this may contribute to an identity crisis for patients (linked to the theme of significant negative impacts linked to loss): if they are not engaging in medical treatment for a medical problem, then they either are a “junkie” or have a mental health problem (“it’s all in my head”.

##### Lack of Coordination and Integration of Care

Patients reported seeking many different types of treatments and supports for their pain. However, there was little reported integration or coordination of care between different providers, the public and private sectors, or traditional and alternative treatments. One participant shared a positive experience with a multidisciplinary rehabilitation program:

It was a triple pronged approach with having the psychologist, the physiotherapist and the exercise physiologist. That was really good.

PID_P

Another participant who was referred to the pain clinic by their GP was not receptive to an integrated approach: “the guy saying ‘you’ll see physio and you’ll see psychologist and you’ll see this and you’ll see that’ and I’m thinking I’ve tried that, been there, done that” (PID_A).

#### Theme 2: Limited Biopsychosocial Understanding of Pain

##### Focus on Physical Aspects of Pain

Most participants focused on the physical aspects of pain and minimized the potential role of psychosocial factors in exacerbating pain. One patient (PID_P) said, “Well I know where my pain is, my pain is a deterioration of the bones...so I don’t think it’s psychological.” There was also reference to experiences where one participant stated, “mine is physical pain not psychological pain” PID_P), with another participant describing pain as being perceived as separate from the mind:

Like if you’re in pain, how can your mind tell you that you’re not in pain? That’s how we’re looking at it. As if to say, well, you’re going to go get pain management, and they’re going to psychologically move your brain around to say that you’re not really in pain, you don’t really need these tablets.... but that still doesn’t have to do with my pain. And I try and keep the two separate because pain is one thing, and keeping my mind sane is another thing.

PID_A

Some participants did describe how psychosocial factors influenced their pain experience. This knowledge often emerged in hindsight or in a precontemplative state of considering treatment options other than opioids. Examples of the possible role of biopsychological factors were often inconsistent or lacked detail. For example, one patient (PID_P) said, “I think there is a big mental side to it as well.” Overall, patient connection with social influences was limited.

##### Disconnect Between Mental Health Problems and Pain and Engagement in Psychological Treatment

Several people described having a co-occurring mental health problem (eg, depression, anxiety, and posttraumatic stress disorder) for which they were receiving treatment from a psychiatrist or psychologist. This was also reflected in the clinical characteristics of the sample. Despite this, there was little evidence that they perceived their mental health as influencing the levels of pain experienced or vice versa.

Participants did not describe accessing psychological treatment specifically for pain or mention that psychological treatments were recommended. One participant (PID_P) reported:

With only recently going to the pain management sort of introduction that they told us that psychiatrist can...psychologist can sort of discuss pain with you and I just didn’t know that that had anything to do with being on pain killers....I think it’s fantastic because I think the brain is a powerful thing and I saw what cognitive behavioural therapy did with my son.

Some patients reported positive experiences with psychological treatment for mental health problems but did not link how psychological treatments could be potentially effective for pain.

#### Theme 3: Continued Opioid Use When Benefits Do Not Outweigh Harms

##### Overview

Participants reported relatively more risks and consequences (eg, side effects and limited pain relief) versus benefits of their opioid use, but they continued to use them. Patients said the following:

I would reduce it down to nothing if I could...because I don’t like taking them or just the way it makes me feel.

PID_P

Well if I could maybe reduce some, it would be good, But I can’t see it happening.

PID_P

Patients also described frustration about taking opioids for pain, while at the same time, they reported holding out hope that there may be other options (eg, “it’s a catch 22...I have to think clearly if there’s a better way of doing it, than to be popping pills every day, then I’m open to anything” [PID_P]).

##### Opioids Mask the Pain

Some patients in this sample reported that opioids did not provide effective pain relief, but this varied from person to person; for example, “It’s not like taking painkillers is actually stopping or making the pain better” (PID_P) and “(opioids) doesn't knock all the pain over” (PID_A). There were reports that opioids “take (pain) down a few notches” (PID_P); “take the edge off” (PID_A and PID_P); or “drowns out the pain” (PID_P). Others did report that medications got rid of the pain (PID_A) and that opioids work better than other medications in the market (PID_A).

In the cases of limited pain relief, opioid medications were often not reportedly taken as prescribed (theme 4): “...even the opioids aren’t really helping with this pain. So that’s why I haven’t been taking them religiously, like I probably should have been” (PID_P). Another participant said, “it just puts me to sleep, I don’t feel anything...I don’t get the pain relief from the pill, you get pain relief from sleep” (PID_P).

##### No Options Other Than Opioids

Most participants reported that they had no other option but to use prescription opioids for pain. One patient (PID_P) said, “I can’t not take it, I don’t have any other option”; another patient (PID_A) said, “But with me, I’d rather not have them myself, you know, I don’t know any other way you can deal with the pain when an operation has gone bad.” For some patients, there was a view that they had no choice but to take opioids, which was related to feelings of frustration.

##### Escalating Dose and Need for Opioids

Participants described a history of escalating dose over time. Patients said the following: “it was two, then four, then six then eight Panadeine Forte...my body had totally adjusted to them...my body was saying, yep I’m having more and more of those” (PID_P); “That’s the first time I was put on an opioids, Endone think it was, and then started off through there, and as the pain got worse over the years the medication got higher” (PID_A). Patients often initiated dose increases with their health professional. One patient demonstrated awareness of this: “because in the long-run I’m going to need a detox clinic, then I’m going to need someone from this clinic...psychologists and psychiatrists” (PID_P)*.* Some patients may have been experiencing withdrawal: “sometimes I just forget my lunchtime dose and what reminds me is the severe drop in mood” (PID_P). Continued use despite negative consequences (eg, social, physical, or psychological problems) and features of tolerance and withdrawal were also reported.

#### Theme 4: Trial-and-Error Approach to Opioid Use

Some patients reported that they were taking opioid medicines as a “trial-and-error” approach to get relief. This appeared to be related to efforts to take control of their pain. Some people said that they took their medication when they were in pain rather than as prescribed:

I try not to take the opioid if I can help it. Wait to see how bad the pain gets before I take it (opioids)...the pain is unbearable. So, I get to the stage where nothing’s helping.

PID_P

I do try to do without them and as long as I possibly can and then sometimes, the pain is so bad...then waste half a day in bed, I get pissed off at myself about it.

PID_P

These reports reflected patients’ delayed use of opioids when in pain to minimize reliance on opioid therapy; that is, patients were hoping or taking the risk that their pain would not become more severe, so they did not have to use opioids.

Others described overuse of opioid medications:

Take more... Yeah. Yeah. I have four of what he gives me for that day and then there’s one for the morning...My overuse of Oxycontin.

PID_A

Some patients reported a history of overuse: “Yes, it was taking too much Targin and before that it was too much morphine, I was actually on morphine and I was taking too much of it” (PID_A). Opioids were also used for other reasons such as sleep or improved mood. For instance, a patient (PID_P) reported the following:

It just puts me to sleep, I don’t feel anything...I don’t get the pain relief from the pill, you get pain relief from sleep.

Patients also reported using stockpiled opioid medications:

I had some old medication that I had from old surgeries...so I would take them when my pain was like 9 out of 10.

PID_P

There were also examples of patients overusing other medicines (eg, benzodiapines and antiepileptics) in conjunction with opioids when they were in severe pain. There were examples where patients decided to self-taper their opioids, without health professional support:

I tried to go cold turkey initially and that was shocking...I thought I would try and reduce on my own.

PID_P

#### Theme 5: Cycles of Hopefulness and Hopelessness in Desperation to Find Pain Relief

##### Overview

Patient reports reflected cycles of hopelessness and hopefulness associated with pain treatment in a desperate effort to find relief. Those with increased pain or pain moving to different parts of the body over time reported a sense of hopelessness about the future: “In the last 3 years, it’s gotten worse and I don’t know what else is going to work” (PID_P). Desperation to find pain relief was commonly reported among participants, which was reflected in the sheer number and variety of other strategies tried and tested over time. This theme linked to the unknown cause of pain and uncertainty that prompts engagement in help-seeking behaviors to either find the source of pain or physical treatments or other strategies to relieve pain (theme 6). While patients tended to use less treatments or strategies over time due to feelings of defeat or failure, burden of treatment, and financial reasons, some seemed to be always looking for the next treatment opportunity.

##### Cycle Back to Previous Treatment Options

Patients described a pattern where they engaged in various pain treatments (eg, physiotherapy and chiropractic treatment) often earlier in their pain journey and that it did not help or became increasingly less effective. This was associated with feelings of hopelessness and defeat, resulting in them taking a break from treatment or trying other strategies until they were prompted to engage in physiotherapy again. One patient (PID_P) reported as follows:

To be told, I want you to go back and do physio and then stay on tramadol and come back in 10 months.... I have already been through all of that.

Some patients reported significant frustration that went along with what they assessed as ineffective treatments.

##### Seeking New Treatments Despite Hopelessness

Participants appeared to hold feelings of hopelessness about current pain treatment and hopefulness that something still may help in the future, at the same time. Some patients were optimistic for a cure and still wanted to be “fixed.” One patient (PID_P) reported, “all I’m asking for is just to get fixed and just go back to work and get my life back on track.” Patients said that hope tended to decrease over time, with one participant reporting, “I was definitely more hopeful earlier on, I’m just thinking there’s going to be something that’s going to be sorted out” (PID_P). Despite this, some patients were open to the idea of receiving psychological treatments as well as medical or surgical interventions or alternative treatments (eg, stem cell therapy) in the *hope* that this may relieve their pain.

#### Theme 6: Diagnostic Uncertainty and Associated Help-Seeking Behaviors to “Fix”

This theme reflects the participants’ uncertainty about the underlying causes of pain which were varied and often unexplained, contributing to ongoing attempts to understand the origins of their pain. Distress, anger, and a sense of unfairness was often tied to the triggering pain event or onset of pain, which was still apparent many years later.

For some individuals, the main explanations for the originating causes of their CNCP were seemingly unusual and involved either internalization (eg, diet and posture) or externalization (eg, weather and surgical or medical interventions going wrong) of blame. For others, there was no known cause for primary pain and patients had difficulty pinpointing the triggering event to their CNCP condition. One patient (PID_P) reported, “I couldn’t tell you because it has always been there on one level, but over the course of the years it’s gotten to the level where it is now.” Diagnostic uncertainty contributed to ongoing attempts to understand their CNCP (cause) and find a “cure” (treatment) through more assessments and tests (eg, “...let’s see what happens with this MRI” [PID_A]). Help-seeking behaviors were associated with hopeful or hopeless feelings about pain treatments and their effects (theme 5). Particularly, in cases where medical diagnoses did not explain the cause of pain, this resulted in patients being in a constant holding pattern, waiting for treatment or further tests.

#### Theme 7: Significant Negative Impacts Linked to Loss

Participants reported a significant number and range of negative impacts of their pain and pain management. These impacts were biological and physical (eg, dental problems); psychological and functional (eg, cognitive and memory problems, sleep disturbance, difficulties engaging in daily tasks, and low mood); or social and economic (eg, social isolation, financial burden of treatment, can’t drive, and intimacy and relationship problems) in nature. One participant (PID_A) spoke about pain as a disability. Another shared that these impacts were often centered around grief and loss: “Most days I feel like I’m letting everybody down in my family because I’m not out there making money” (PID_P). The interviewer observed that participants become the most distressed in the interview when they compared their life before the pain to how it is now—and what they had lost or had to try to change or adapt. This was interpreted as a sense of loss of participating in life and normality.

For some, this sense of loss was also related to a loss or changing of identity:

If I come off the 10 tablets, am I going to be in more pain or am I going to be in the same pain as I am now...but have me back I’m me, have me back, just be myself.

PID_P

This included changes to their identity over time—to a “chronic opioid user” or “addict.” Others noted a more recovery-focused interpretation of their identity with opioid use. A patient (PID_A) said the following:

Well, that’s just part of what you did. It’s not who you are. It’s what you do. It doesn’t have to define you. You can live a normal freaking life, with or without opioids. You’ve got a choice.

Importantly, the varying ways people used prescription opioids (theme 4) was interwoven with these impacts; that is, the use or misuse of opioids was often to reduce the impacts of pain on their life.

#### Theme 8: Complexity of Pain and Opioid Use Journeys

Participants described complex pain journeys that involved variations in pain experience over time (got better or worse) and either a slow or fast track to opioid use. The uniqueness of the pain experience was also highlighted.

##### Slow Versus Fast Track to Opioid Use

The slow track to opioids often started with physiotherapy and primary care efforts to relieve pain and, in some cases, for specialists to “fact find.” Over the course of their pain journey, there was a common feeling that patients were exhausted trying to investigate the physical or organic cause of pain. Because of failed treatments, patients were often initiated on opioids. One patient (PID_P) said, “cause he’s the one that ... organised me to go to physiotherapy and all this, and that’s when it didn’t work. That’s when I really started on this, using that.” This pathway was common among patients from the pain clinic.

For other participants, a fast track to prescription opioids was initiated as part of acute pain management, associated with acute injury or surgery. These individuals were initiated on opioids in hospital and on discharge. A patient (PID_A) described as follows:

Pretty much straight away when I was in pain (in hospital).... Yes, they gave me medication to take home and then take the deep heat and that’s how it started.

Participants described either positive or negative experiences of being prescribed opioids for the first time. Some patients reported immediate pain relief or euphoria. This sense of relief was reported as sometimes occurring after previously trying multiple types of opioids. Other patients described experiencing adverse side effects and limited longer-term pain relief:

I knew a little bit about it but it didn’t really deter me from it because, you know, not going to work, not paying bills, seems more of a downside than just taking something throughout the day that can help you continue on with your life.

PID_A

##### Everyone’s Pain Experience Is Unique

Patients described the experience of pain as unique to each individual. One participant (PID_A) said the following:

Everyone is different... Because it’s a very individual thing, I think. Handling pain, dealing with pain.

Additional statements from the workshops provided further validation of this subtheme (eg, “well I’ve always had pain...pain is a personal thing” [PID_P]).

### Interviewer Observations

During the patient interviews, there was evidence that some patients benefitted from the interactions with the interviewers (psychologists). This was evident even for those patients who reported that it was really challenging to describe their pain journey and they would not have been able to do this if it was years ago. There was a view that listening, asking nonjudgmental questions, and showing empathy resulted in some patients softening throughout the interview when they appeared to be more defensive at the outset:

You went about it the correct way. I used to get annoyed at people quite easily. So yeah, you done it fine, you ask questions nicely, you didn’t talk over, you stopped when someone else is talking, which is a nice thing. So, you conducted it very well so it made it a lot easier.

PID_A

Alternatively, for one patient (PID_A), the interview brought attention to past pain experiences, and this was related to an increased experience of pain: “Like my backs starting to hurt just thinking about it.”

There appeared to be increased interest in understanding and openness to the biopsychosocial model at the end of the interview (eg, compared with starting the discussion around the aim of the study). Some patients appeared to reflect upon and repeat the points on the biopsychosocial model throughout the interview.

### Validity Checking

The process of member checking resulted in several changes to the theme structure. These included the following:

1. Tapering fears and lack of patient-physician partnering was integrated in theme 1. Furthermore, lack of coordination and integration of care among health professionals; influence of policy change judgment; and mistrust of medical profession were also included in theme 1.
2. We removed seeking certainty for cause of pain and looking within and looking outward for explanations; these were absorbed in theme 6. Furthermore, we refined the theme to focus on diagnostic uncertainty and associated help-seeking behaviors to capture these elements.
3. Theme 8, previously named multiple pain journeys and pathways to opioid use, was reconceptualized as complexity of pain and opioid use journeys. This was to capture the complexities of pain management (either fast or slow track to opioids) and also the unique experience of pain (a subtheme that emerged from workshop 1).

## Discussion

### Main Findings

This study is one of the first to describe the lived experience of individuals with CNCP in the context of co-designing a digital BI to increase the safe use of pain medicines. In the sample of patients interviewed, half of the patients met the current opioid misuse thresholds on validated psychometric scales (COMM [57]). Our main findings were the following 8 overarching themes that emerged in this sample related to the lived experience of patients with CNCP regarding pain management including opioid therapy: limited treatment collaboration and partnership; limited biopsychosocial understanding of pain; continued opioid use when benefits do not outweigh harms; trial-and-error approach to opioid use; cycles of hopefulness and hopelessness; diagnostic uncertainty; significant negative impacts tied to loss; and complexity of pain and the opioid use journey.

Overall, patients felt they had limited choice but to continue opioid therapy, as there were few perceived alterative treatment options available, a common concern also shared by physicians [64]. Patients’ fears about making changes to their opioid use may have also played a role. This state of “stuckness” may have contributed to patients’ sense of hopelessness about pain and its management. In many ways, patients may be rightly consumed by their pain in a way that they may be ambivalent about many aspects of pain management and life in general. Opioid therapy was also linked to behaviors that could elevate patient risk (eg, not taking medication as prescribed, self-tapering, and combining different opioids and classes of drugs) in a trial-and-error approach. Opioid use generally provided a pain “mask” with limited pain relief, sometimes; consistent with the results from meta-analyses [4]. However, some patients from addiction services described more positive experiences of opioid medications. Altogether, these experiences signal gaps in our current models of care and suggest that BIs may provide an avenue for (1) patients to explore thoughts and feelings about opioid use, (2) treating clinicians to support patient-physician partnering, (3) opioid use education (risks and harms), and (4) exploring patient ambivalence (risks versus benefits of status quo versus change) as a precursor to resolving the push and pull of behavioral change. Whether these techniques translates into behavioral change requires further testing. Certainly, there is evidence for the effectiveness of BIs in reducing substance-related harm [26,27] and growing evidence for their role in treating pain [65].

BIs provide another avenue to improve patient understanding of the biopsychosocial model of pain, critical to effective pain care [66]. The biopsychosocial model is broad and complex, and our explanation during the interview was brief and over the telephone. While participants may have received some education in the past, it was not obvious during the interview that they had a clear prior understanding of the influence of physical and psychosocial factors in the development and management of pain. When discussing the biopsychosocial model as a part of member checking, the use of visual tools to explain the concepts, the group peer setting, and reinforced messages may have contributed to patients’ more positive responses to the model. Educating patients in the biopsychosocial model of pain is an important part of multidisciplinary rehabilitation and discipline-specific treatments that can be effective (eg, [67]). Efforts to enhance the education of health professionals in the biopsychological model could be important in contributing to improved patient understanding [68-70]. The consideration of patient health literacy and eHealth literacy [71] could help tailoring pain education to patient needs. These approaches are especially important, given that the lack of understanding of the biopsychosocial model may be related to ongoing diagnostic uncertainty and attempts to find a (biological or physical) source of pain and treatments that “fix.”

Patients indicated that specific psychological treatment for pain was rarely offered, although many patients had previously engaged with psychologists or psychiatrists for other co-occurring mental health reasons. These reports are consistent with survey data from the United States indicating that 37% of patients are not aware of this treatment option and only 33% had ever worked with a pain psychologist [69]. This is despite psychological treatments being one of the recommended evidence-based treatment options for the treatment of chronic pain and in conjunction with opioid therapy [3,8,72]. Approaches that raise awareness and provide education on the role of psychology in the treatment of pain are clearly needed not only at the patient level but also at the provider and public health systems level [73]. Most patients reported being open to this approach. Therefore, it is necessary for the psychologist workforce to meet this need for psychoeducation [13]. This will be especially important, given the psychologist workforce shortages and growing demand in the post–COVID-19 pandemic era [74].

Patient–health care professional communication and relationships had a significant impact on patients’ emotional well-being and in seeking appropriate pain care. Patients felt that various aspects of opioid therapy (eg, monitoring, dose reduction, and policy change) could have been managed differently. It is acknowledged that we do not know about the patient’s prescription instructions or if there was a management plan to taper them (as recommended in clinical guidelines). Our findings suggest that partnerships could be optimized by enhancing the patient–health care professional alliance and shared decision-making. Assuring patients that they will not be neglected by the treating practitioner in their medication management is essential [75], as is the need to make patients feel heard and understood. There were several examples of effective patient–health care professional communication and effective multidisciplinary management of the patients’ pain and health concerns. Our study found that these examples of patient care were positively received by patients.

Questions in our interviews explored the patient’s pain journey, including participation in psychological treatments as well as the pros and cons of opioid use, consistent with a motivational interviewing framework. Applying patient-identified motivational BI strategies may give patients with complex chronic conditions a space to share and feel heard. This can often be the first important step toward facilitating behavioral change [76,77]. Motivational interviewing BI techniques might also be particularly beneficial in exploring ambivalence, that is, teasing out where adverse effects and consequences of opioid use outweigh the benefits (ie, positive effects on pain or daily functioning); pain triggers; different pain management strategies; or highlighting unintended consequences to those who do not know they are at elevated risk of opioid-related harm.

### Recommendations for the Design of BIs

On the basis of our research findings, we provide 6 core recommendations (not in any particular order) for co-designing and codeveloping digital BIs. Textbox 1 presents a summary of the recommendations. Future research should consider the potential barriers and enablers to implementing these recommendations during the design and development process. For example, patients may be reluctant to consider psychological therapies or to see a psychologist (recommendation 1), as their pain experience may be perceived as “all in their head.” Health professionals (especially GPs) may believe that they do not have enough time to address aspects of pain management, such as the ups and downs of the pain experience and pain treatments (recommendation 2), given the demands of primary care.

**Summary of recommendations for the co-design and co-development of digital brief interventions.**

**Recommendation 1: educate about the role of psychologists and behavioral therapies in pain care**

Educate patients and health professionals about the role of psychologists and mental health professionals and the evidence and clinical recommendations for use of psychological therapies in pain care. This is necessary to ensure there are no missed opportunities for patients to access psychological treatments earlier in their pain journey or as a first-line treatment.

**Recommendation 2: normalize the hopefulness and hopelessness cycles in pain treatment**

Normalize the cycle of pain treatment and associated feelings of hopefulness and hopelessness in the proposed brief intervention (BI) by discussing expectations and potential challenges, such as the idea of setbacks. Informing patients that they may go back to strategies that they have tried before may be helpful. The degree to which they are experiencing hope and optimism about treatment could be acknowledged and explored. Sharing what works with peers could enhance pain self-efficacy and social connectedness.

**Recommendation 3: communicate with compassion and empower patients in patient–health care professional partnerships**

Incorporate key principles of partnership and shared decision-making between health professionals and patients. Inviting patients to share their story could strengthen patient–health professional relationships as well as acknowledging feelings of loss and frustration. Strategies to empower patients and communicate with compassion are needed. Regular monitoring and feedback of opioid therapy by health professionals may assist patients. Digital solutions could also optimize this goal.

**Recommendation 4: inform about the risks of unsafe opioid use and explore any ambivalence**

Weighing the individual benefits versus harms of current opioid use may assist in exploring ambivalence about change. Educate patients about the possible harms of opioid use and contraindications. Developing insight into potentially unsafe opioid use patterns may be aided by monitoring, assessment, and feedback. Motivational interviewing may provide a guiding framework.

**Recommendation 5: educate about the biopsychosocial model of pain**

Educate patients about the biopsychosocial model of pain in a simple and engaging way; that is, the dynamic interaction among physiology, psychological, and social factors that can contribute to and perpetuate pain. Provide textual and visual cues and supporting materials to aid explanation. It will also be important to address ongoing patient efforts to seek a medical or physical explanation for the onset and maintenance of pain. A clear definition of psychological treatments is also required as well as consideration of marketing of the BI, particularly when patients have a history of failed pain management strategies.

**Recommendation 6: acknowledge stigma and the uniqueness of pain and personalize management to different care pathways**

Acknowledge stigma and personalize management to different care pathways (eg, slow or fast track to opioids and different referral or service perceptions). Patients with pain see themselves as different from other patients of addiction services. Notwithstanding, patients from pain clinics also expressed that they experience stigma related to chronic noncancer pain and opioid use. Acknowledge the uniqueness of each pain experience. Tailoring of content may also be required. Distinguishing concepts, such as addiction versus physical and psychological dependence, may also be important. Consideration of recovery-focused language may assist in exploring impacts of diagnostic labels on identity (internal or social conflicts; sick role).

### Strengths and Limitations

Several strategies were used to increase the generalizability of the findings to the complex pain population. A broad sampling strategy was included to capture diversity, including age, culture, language, sex, and history of opioid therapy. Despite these attempts, culturally and linguistically diverse patients were not well-represented. Ensuring cultural diversity is incorporated as much as possible in the subsequent steps of co-design and co-development of BIs could increase customizability of the solution to fit a wider range of users. Our strategy also considered the sample size required for data saturation prospectively during data collection using a new assessment method [52]. The validity of the themes and subthemes was further enhanced through patient member checking. Future research could explore whether these core themes generalize to individuals living in a community (not actively seeking specialist treatment) in a replication study. Patients from specialist addiction and pain clinics were recruited using different strategies. This was necessary to integrate recruitment into the existing models of care, but it may have affected initial patient engagement. Patient reports may have also been influenced by their position in the treatment pathway when they were engaged in this study (eg, before multidisciplinary pain clinic care vs during the initial visit vs review visit). Our study was conducted in Australia, where patients have access to a relatively high standard of public health care and access to heavily subsidized medications. Investigations in countries with different health care systems are needed. There remains a power differential between patients, health care providers, and researchers. It is unknown how this may have influenced our findings. Future work could acknowledge and explore relationship power imbalances in the initial design steps and jointly develop guiding ethical principles. Inviting patients to be members of the research team will be important.

### Conclusions

BIs have a long history in chronic relapsing conditions [24,25]. It remains to be discovered whether this treatment can be successfully applied to patients with CNCP who are at risk of opioid-related harm. However, we have made progress in effectively engaging *patient partners* in the co-design of a digital BI, the first necessary step. Exploring lived experiences as part of contextual inquiry could be considered a predesign step to co-design [78]. Our findings add to the growing literature on pre- or co-design in DHIs [79] and extend research into the CNCP field. Co-design has advantages in designing fit-for-purpose solutions that have the potential to enhance treatment effectiveness, adoption, and reach for individuals with CNCP at risk of prescription opioid–related harm.

This important first step of partnering with patients who have CNCP to understand their lived experience is integral to the co-design of new and innovative digital psychological treatments. Without this, many of the challenges identified by patients (eg, lack of trust and accessibility of treatment options) would compromise later co-design phases. The next phase in co-designing personalized digital BIs involves the generation of early design concepts and prototyping, by bringing together scientific, patient, clinical, and technical expertise to meet our current and foreseeable complex challenge of patient-centered, safe opioid therapy in CNCP care.

## Appendix

Multimedia Appendix 1 Patient semi-structured interview questions.

Multimedia Appendix 2 Sample characteristics of patients.

## Abbreviations

BI: brief intervention
CNCP: chronic noncancer pain
COMM: Current Opioid Misuse Measure
DHI: digital health intervention
GP: general practitioner

## Data Availability

The datasets generated during and analyzed during this study are not publicly available due to limitations of ethics approval but are available from the corresponding author on reasonable request.
