# Supplementary material for: Co-Designing a Digital Brief Intervention to Reduce the Risk of Prescription Opioid–Related Harm Among People With Chronic Noncancer Pain: Qualitative Analysis of Patient Lived Experiences
Source: JMIR Form Res. 2025 Jan 30;9:e57208. doi: 10.2196/57208 (PMC11826952; doi:10.2196/57208)
Supplement: Multimedia Appendix 1 [file formative_v9i1e57208_app1.docx]

**Multimedia Appendix 1 - Supplementary Materials**

Table S1. Patient semi-structured interview questions.

| Semi-structured interview questions |
| --- |
| **Introduction**  **Opening question:** Describe a typical day of how you manage your pain. Start from the moment you wake up.  **Pain Experience and Management**   1. What is your pain level right now, scale 0 (no pain at all), to 10 (the worst pain you can imagine)? [acknowledge pain level and check if ok to proceed with interview] 2. In general, what pain management strategies are you currently using? 3. What works with how you are managing your pain now? What doesn’t work? 4. How well do you think you are managing your pain at the moment? 5. What help, supports, or services are you currently accessing for pain? 6. One of the main explanations of pain, is that it is caused by a range of physical, social and psychological factors. This includes physical changes within your body – tissue or nerve damage for eg; psychologically, such as the thoughts you have about your pain and how you feel; and the social environment such as your level of social support. What do you think about this as an explanation of pain?    - - 1. How does this explanation relate to your experience of pain?        2. Do you think your pain is specifically focused in one of these areas or a combination of some/many?   **Opioid Medication Use Experiences**   1. Using prescription opioids is one way to manage pain and is sometimes a way that people manage their pain. I asked you about your opioid medications last time we spoke [acknowledge opioid medication use and any related experiences]. In general, what’s good about using prescription opioids? In what ways is it working for you? What’s not so good about using prescription opioids? In what ways is it not working for you? 2. How well do you think you are managing your opioid medication regimen at the moment? 3. Overall, how do you feel about how you are managing your pain with opioids? 4. What are some of the reasons that you use opioids? 5. One the reasons people might use opioids is to manage their pain. Would you say using opioids effectively manages your pain? Why/why not? 6. Managing some of the unintended side effects of opioids such as… constipation, dizziness, dry mouth, headache [add those relevant to the patient] can be difficult. How do you go with this? 7. Some people sometimes find it difficult to take their medication as prescribed. How do you go taking your opioid medication as prescribed? 8. Sometimes people can take too much or too little prescription opioids and have difficulty following their Dr’s recommendations. Have you ever experienced this? [link to past responses if relevant] 9. Are there any challenges for using them in this way? 10. We have discussed reasons that you use opioids. These included [list from previous response]. Are these the same as why you might sometimes not take your medicine as prescribed? How are they similar or different?   **Role of pain medication beliefs and expectancies and other psychological factors**   1. We asked you to complete a few questionnaires about your beliefs about pain and pain medications. I’d like to ask you a few follow up questions if that’s ok [refer to questionnaire responses; examples below about how these questions were framed]. 2. One of the items was “I will probably always have to take pain medication”. You indicated that this was eg “very true of you”. Can you tell me a bit more about why you think that you will probably always have to take pain medication? 3. In another question you mentioned that you don’t think your pain will decrease in the next month (ie <50%). Can you tell me a bit more about why you don’t think it would decrease? 4. Sometimes higher levels of stress, anxiety, or depression/ or negative thoughts about pain can make pain or opioid medication use worse; have you ever experienced that?   **Treatments and Help Seeking from Health Services Specific to Opioid Use**   1. I’m interested to know what specific programs or interventions you might be accessing at the moment in relation to your opioid medication use. What is helpful? Unhelpful? 2. There are services available to help people make changes to their opioid use. Some people might be supported by their pain or addiction specialist or GP for example [Consider if patient is on ORP]. Are you currently making any changes to your opioid use regimen? What type of changes and why? 3. People’s safety is really important when taking opioids. You mentioned that you sometimes may not currently take your opioids as prescribed. Are you currently seeking any help for this? 4. A few people engage in programs to help them manage their opioids safely or to reduce their use. What specific programs or interventions if any are you currently participating in to reduce your opioids or help you take them as prescribed? 5. Sometimes people might participate in psychological therapy such as cognitive behavioural therapy when making changes to their opioid use. Clinical guidelines also recommend this. Are you currently participating in any psychological interventions? Why? Why not? 6. There are lots of different ways to deliver services & programs. Are you currently engaged in any services or programs that use telehealth/online?   **Impacts**   1. Pain and sometimes opioid use can have a big impact on all areas of \life. How does your pain level and/or opioid use currently impact on your social life? (friends, family, social activities)? Work? Home? Spiritual? Cultural?   ***Past and Future Questions**  Patients were then asked similar questions but were asked to consider their experiences over time.   1. Now I am going to ask you some similar questions, but I would like you to consider the past 12 months compared to now/today. Has your typical day been any different over the past 12 months? 2. Now I am going to ask you some similar questions, but I would like you to consider the time you first experienced pain. Has your typical day been any different since then? 3. What about the time you were first prescribed opioids. Has your typical day been any different since then compared to now? |
|  |
