# Supplementary material for: Co-Designing a Digital Brief Intervention to Reduce the Risk of Prescription Opioid–Related Harm Among People With Chronic Noncancer Pain: Qualitative Analysis of Patient Lived Experiences
Source: JMIR Form Res. 2025 Jan 30;9:e57208. doi: 10.2196/57208 (PMC11826952; doi:10.2196/57208)
Supplement: Multimedia Appendix 2 [file formative_v9i1e57208_app2.docx]

**Appendix 2**

**Table S2.** Sample Characteristics of Patients

| PID | Age (yrs) | Sex | Ethnicity | Pain location^a^ | Pain duration (years) | DASS-D | DASS-A | DASS-S | Pain Interference (past week) | Pain Severity (past week) | COMM | Opioid use duration (years) | OME |
| --- | --- | --- | --- | --- | --- | --- | --- | --- | --- | --- | --- | --- | --- |
| PID_P | 33 | F | Australian | Back | 15 | 0 | 2 | 0 | 2.71 | 2.25 | 4 | --^g^ | 1.15 |
| PID_P | 52 | M | Australian | Back, lower limbs, abdomen | 2 | 16 ^e^ | 0 | 16 ^d^ | 9.14 | 7 | 5 | 1.25 | 30 |
| PID_P | 60 | F | Australian | Neck, upper limbs | 3 | 4 | 4 ^b^ | 12 ^c^ | 5.00 | 6 | 7 | 2 | 45 |
| PID_P | 31 | M | Australian | Back | 5 | 14 ^e^ | 6 ^c^ | 16 ^d^ | 4.14 | 5 | 14^f^ | 3 | 60 |
| PID_P | 47 | F | Australian | Head, neck, abdomen | 0.5 | 14 ^e^ | 12 ^e^ | 18 ^e^ | 4.71 | 6 | --^g^ | --^g^ | --^g^ |
| PID_P | 61 | F | Australian with English Irish Decent | Upper limbs, back | 4 | 2 | 4 | 4 | 2.86 | 5 | 8 | --^g^ | --^g^ |
| PID_P | 53 | F | Welsh and Australian | Head, neck, back, abdomen | 14 | 0 | 8 ^d^ | 10 ^c^ | 4.71 | 4 | 10^f^ | --^g^ | 70 |
| PID_P | 47 | F | Australian | Upper limbs, back | 0.83 | 4 | 10 ^e^ | 2 | 6.57 | 6.75 | 20^f^ | 0.75 | 22.8 |
| PID_P | 58 | F | Australian | Whole body | 42 | 26 ^e^ | 16 ^e^ | 32 ^e^ | 8.86 | 8 | 13^f^ | 20 | 143.4 |
| PID_A | 60 | F | Australian | Lower limbs | 2.5 | 4 | 0 | 6 | 6.43 | 7.25 | 2 | 2.5 | 37.9 |
| PID_A | 54 | M | Australian | Back, whole body | 20 | 30 ^e^ | 26 ^e^ | 32 ^e^ | 7.43 | 7.25 | 25^f^ | 22 | 480 |
| PID_A | 25 | M | Australian | Back | 3 | 4 | 12 ^e^ | 14 ^d^ | 5.83 | 6.5 | 1 | 0.58 | 4.2 |
| PID_A | 58 | M | New Zealander | Head, lower limbs | 11 | 2 | 10 ^e^ | 10 ^c^ | 6.43 | 5.5 | 15^f^ | 11 | N/A^h^ |
| PID_A | 53 | M | Australian | Neck, upper limbs, back, lower limbs, whole body | 20 | 12 ^d^ | 6 ^c^ | 14 ^d^ | 5.29 | 5.75 | 13^f^ | 10 | N/A^h^ |
| PID_A | 56 | F | Greek | Head, neck, whole body | 28 | 12 ^d^ | 8 ^d^ | 12 ^c^ | 8.00 | 6.75 | 3 | 26 | N/A^h^ |
| PID_A | 48 | M | Australian | Upper limbs, back | 9 | 10 ^c^ | 0 | 8 ^b^ | 4.14 | 6.5 | 11^f^ | 8.5 | 90 |
| PID_A | 33 | F | Australian | Neck, upper limbs, back, whole body, abdomen | 3.5 | 22 ^e^ | 34 ^e^ | 26 ^e^ | 4.29 | 3 | 27^f^ | 3 | 480 |
| PID_A17 | 62 | M | Australian | Back, abdomen | 25 | 18 ^e^ | 8 ^d^ | 18 ^e^ | 7.71 | 6.25 | 16^f^ | 2 | 20 |

*Notes.* ^a^ head = head/face, upper limbs = shoulders/upper limbs, back = back/spine/sacrum, abdomen = abdomen, pelvic, genitals, buttocks, groin, ^b^ mild score, ^c^ moderate score, ^d^ severe score, ^e^ extremely severe score, ^f^score has met the cut-off for the scale as risk of unsafe opioid use, ^g^ missing, ^h^Patients were using methadone and dose could not be calculated. K6 = Kessler Screening Scale for Psychological Distress 6; BI = Brief Pain Inventory; DASS-D = DASS-21 Depression subscale, DASS-A = DASS-21 Anxiety subscale, DASS-S = DASS-21 Stress subscale, COMM = Current Opioid Misuse Measure, OME = Oral Morphine Equivalence (daily). PID_P refers to patients from the pain clinic and PID_A refers to patients from specialist addiction services.
